# Difficult septal deviation cases: open or closed technique?^☆^

**DOI:** 10.1016/j.bjorl.2016.03.015

**Published:** 2016-04-29

**Authors:** Sultan Şevik Eliçora, Duygu Erdem, Hüseyin Işık, Murat Damar, Aykut Erdem Dinç

**Affiliations:** Zonguldak Bülent Ecevit University, Faculty of Medicine, Department of Otorhinolaryngology, Zonguldak, Turkey

**Keywords:** Nasal septum, Nasal surgical procedures, Intranasal surgery, Septo nasal, Procedimentos cirúrgicos nasais, Cirurgia endoscópica

## Abstract

**Introduction:**

The aim of this study is to compare the functional aspects of open technique (OTS) and endonasal septoplasty (ENS) in “difficult septal deviation cases”.

**Methods:**

60 patients with severe nasal obstruction from S-shaped deformities, multiple deformities, high deviations etc. were included in the study. The OTS was used in 30 patients and the ENS was performed in 30 patients. The Nasal Obstruction Symptom Evaluation (NOSE) scale was administered preoperatively and at first month following surgery. Patients were also evaluated for pain postoperatively with Visual Analog Scale (VAS).

**Results:**

The mean NOSE score was decreased 62.5–11.0 in the OTS group and 61.3–21.33 in the ENS group. Improvement of the symptoms following the two surgical techniques is similar and no statistically significant difference was found between both techniques. Also there was no statistically significant difference in postoperative pain between the OTS and ENS groups evaluated by VAS.

**Conclusion:**

ENS is as successful as the OTS in management difficult septal deviation cases. In patients with severe septal deformities type of the surgical technique should be selected according to the surgeon's experience and the patient's preference.

## Resumo

**Introdução:**

O objetivo deste estudo é comparar os aspectos funcionais da Septoplastia entre a técnica aberta (STA) e a endonasal (SEN) em “casos difíceis de desvio de septo nasal”.

**Método:**

Foram incluídos 60 pacientes com obstrução nasal devido a deformidades em forma de S, múltiplas deformidades, desvios altos etc. A STA foi usada em 30 pacientes e a SEN em 30. A escala de avaliação do sintoma de obstrução nasal (NOSE) foi administrada no pré-operatório e no primeiro mês após a cirurgia. Os pacientes também foram avaliados com Escala Visual Analógica (EVA) para dor no pós-operatório.

**Resultados:**

O escore médio de NOSE foi reduzido de 62,5-11,0 no grupo da STA e de 61,3-21,33 no grupo da SEN. Houve melhora dos sintomas com as duas técnicas cirúrgicas e não foi encontrada diferença estatisticamente significativa entre elas. Também não houve diferença estatisticamente significativa nos graus de dor no pós-operatório que tenha sido avaliada pela EVA entre o grupo de STA e o de SEN.

**Conclusão:**

De acordo com nossos dados, a SEN é tão bem-sucedida quanto a STA no tratamento de casos difíceis de desvio de septo nasal. Em pacientes com deformidades septais graves, o tipo de técnica cirúrgica deve ser escolhido de acordo com a experiência do cirurgião e a preferência do paciente.

## Introduction

Septoplasty is a common procedure in daily ear nose and throat practice. Various methods of surgical treatment are defined in nasal deformities that cause nasal obstruction: endoscopic septoplasty for posterior nasal obstruction, Cottle's septoplasty for septum's luxation and deviation on the premaxilla area, septoplasty with spreader grafts for dorsum cartilage deviations, extracorporeal septoplasty with a new septum cartilage frame for the complex deviations.^1^ The mostly used technique is still the one that defined by Cottle in 1958.^2^

Severe septal deviations, caudal deformities, anterior deviations, S-shaped deviations, high deviations and mid-dorsal abnormalities are the ones that are defined as “difficult septal deviations”. In such cases endonasal septoplasty can be used by some surgeons but also open technique septoplasty can be preferred to increase angle of vision. Both techniques have different limitations that affect their success. In the open septoplasty, the longer duration of the operation and the formation of postoperative columellar incision scar limit the technique.^3^ On the other hand in the endonasal septoplasty, narrow angle of vision and for that more limited intervention area emerges as a disadvantage. In this study we aim to compare the functional results of the open and the endonasal septoplasty techniques in difficult septal deviation cases.

## Methods

This study was designed as a prospective nonrandomized longitudinal study and approved by ethical committee (Number: 2014-119-01/07). All participants signed an informed consent agreement. Patients who were applied to our ENT clinic because of nasal obstruction and diagnosed as nasal septal deviation between September 2014 and May 2015 were classified according to Mladina's classification^4^ (Table 1). Among these patients who have had Mladina type 4, 6 and 7 deviations were included in the study. The patients with insufficient nasal tip support were excluded from the study. Before the surgery, informed consent was obtained from all patients. The columellar incision was explained particularly. Patients who have needed an additional surgery such as adenoidectomy, endoscopic sinus surgery or turbinate surgery were not included to the study. Revision cases and patients whose age was <16 years were also excluded. Open technique was proposed to all patients, and the patients who agreed the open technique were assigned to the open septoplasty group. The patients who rejected the open technique generally because of the incision scar were included in the endonasal septoplasty group. The study was completed when the patient number reached to 30 in each group.

**Table 1:** Mladina's classification of deviated septum nasi.

	Mladina's classification
Type I	Presence of a unilateral crest which does not disturb the function of the nasal valve. It is situated in the area of the valve.
Type II	Disturbance of the valve function is caused by the unilateral crest. Positive Cottle's symptom can be observed after raising of the nostril, which gives a subjective and objective improvement in the nose patency.
Type III	One unilateral crest at the level of the head of the middle nasal concha
Type IV	Defines two crests – one at the level of the head of the middle nasal concha, and the other on the opposite side in the valve area, disturbing the valve functions.
Type V	A unilateral ridge on the base of the septum, while on the other side the septum is straight.
Type VI	A unilateral sulcus running through the caudal-ventral part of the septum, while on the other side there is a ridge and accompanying asymmetry of the nasal cavity.
Type VII	A mix of types from I to VI.

All surgical operations were carried out by the same team. To evaluate the functional results of the operations the NOSE scale (Table 2) was administered preoperatively and at first month following surgery. The NOSE scale is a symptom specific scale, developed by Stewart et al. in which the patients scored five different symptom specific questions, with 0 meaning “not a problem” and 4 meaning “severe problem”. At the end, these answers were calculated with a total score always between 0 and 20. We then multiplied this score by five and completed it to 100. Higher scores mean the symptom severity was higher. The scale was translated into Turkish, and its reliability in the Turkish population was demonstrated by Kahveci et al. by a previous study. To get the baseline NOSE scores, the patients were asked about the nasal obstruction symptoms prior to the operation.

**Table 2:** The Nose Obstruction Symptom Evaluation (NOSE) scale.

Over the past 1 month, how much of a problem were the following conditions for you? Please circle the most correct response						
		Not a problem	Very mild problem	Moderate problem	Fairly bad problem	Severe problem
1	Nasal congestion or stuffiness	0	1	2	3	4
2	Nasal blockage or obstruction	0	1	2	3	4
3	Trouble breathing through my nose	0	1	2	3	4
4	Trouble sleeping	0	1	2	3	4
5	Unable to get enough air through my nose during exercise or exertion	0	1	2	3	4

Midazolam was administered as premedication and surgeries were performed under general anesthesia with Remifentanil and inhalant anesthetic for all patients.

For Mladina type 4 deviations spreader grafts were placed after separation of the cartilage from vomer and nasal crest in open technique. In closed technique an inverted v shaped excision and partial thickness scorings were performed on the posterior concave side of the deviated cartilaginous septum, excision is performed to inferior deviation and relaxation and minimal cartilage excision was applied to anterior deviation.

For Mladina type 6 deviations after elevation of bilateral mucoperichondrial flaps maxillary crest and a small portion of the cartilage was resected. Then cartilage was fixed in midline and suturated to the soft tissue around the maxillary crest in suitable cases.

Combination of these methods was used for Mladina type 7 deviations.

No nasal packing was needed. Bilateral internal silicon splints were used for all patients. For the postoperative pain Diclofenac sodium was used. In order to analyze postoperative pain, the Visual Analog Scale (VAS) was used at the postoperative first day. VAS is a tool by which patients indicated their general satisfaction with the operation, with 1 meaning least and 10 meaning maximum satisfaction on a 10 cm line. Early follow up examinations were performed with anterior rhinoscopy and endoscopic examination on the first and the third weeks postoperatively. Patients were called to follow-up examination and for a survey on nasal obstruction symptoms at the postoperative first month. Patients were followed up at least 6 months postoperatively.

Statistical analyses were performed using commercial software (IBM SPSS Statistics 20, SPSS Inc., an IBM Co., Somers, NY). Two paired sample *t*-test was used to compare the NOSE scores between baseline and post-operative periods. Continuous variables were presented as the mean standard deviation. A *p*-value < 0.05 was considered as statistically significant.

## Results

60 patients were included in the study. Among these patients 30 were treated with the open technique septoplasty and 30 were with the endonasal septoplasty. There were 23 (76.6%) male and 7 (23.3%) female patients with a mean age of 35.2 ± 12.6 in the endonasal septoplasty group. There were 23 (76.6%) male and 7 (23.3%) female patients with a mean age of 38.77 ± 15.8 in the open technique group. The distribution of deviations according to Mladina's classification among two groups was shown in Table 3. There was no statistically significant difference among Mladina's classification between the open septoplasty group and the closed septoplasty group (*p* = 0.688).

**Table 3:** The distribution of deviations according to Mladina's classification among two groups.

	Open technique septoplasty	Closed technique septoplasty	Total
Type 4	5	3	8
Type 6	4	5	9
Type 7	21	22	43
*Total*	30	30	60

In the open septoplasty group the mean NOSE scores at baseline and 1 month after surgeries were 62.5 ± 22.2 and 11.0 ± 13.2 and in the endonasal septoplasty group 61.33 ± 20.38 and 21.33 ± 25.4 respectively. The difference between the baseline and the postoperative scores was highly significant (*p* < 0.001), but the difference between the two groups was not statistically different. In VAS scores for evaluating postoperative pain there was no difference between the two groups (*p* = 0.106). No major postoperative complication (e.g., saddling, recurrence, wound infection, and septal perforation) was seen in any patient. Mild bleeding occurred in one patient at the endonasal group. Minimal synechia occurred in one patient at the open technique group. No revision septoplasty surgery was needed in any patient.

## Discussion

Difficulty in nasal breathing is probably the most common complaint heard in rhinology practice. Among the major causes are nasal septum deviation and allergic rhinitis.^5^ Deviation of the nasal septum can result in nasal obstruction, sinus disease, crooked nose deformity, and other structural problems. Substantial deviations of the nasal septum may also affect the humidification, olfaction, air filtering, and temperature regulation of the nose and finally significantly reduce the quality of life.^6^, ^7^

The best management of the patients with nasal septal deviation is still under debate. There are no evidence-based guidelines for deciding which patients are suitable for surgery, what kind of operation should be done, and which patients will benefit the most.^8^ Especially in difficult septal deviation cases selection of the surgical technique becomes harder. In this study we evaluated the results of the open technique and the endonasal septoplasty particularly in difficult septal deviation cases.

It is hard to choose the exact surgical technique in these cases but it is also harder to evaluate this techniques’ success. In general, evaluable tools to measure the septoplasty results can be categorized as objective, such as rhinomanometry, acoustic rhinometry, computed tomography, and peak nasal inspiratory flow; and subjective, including patient history, the NOSE scale, questionnaires incorporating Visual Analog Scale, the Fairlay nasal symptom score, the Nottingham Health profile, and the general health questionnaire.^5^, ^7^, ^9^, ^10^, ^11^, ^12^ Although no objective method has been validated yet, the NOSE scale developed by Stewart et al. is a promising and reliable method for use in nasal obstruction.^13^, ^14^ This scale's reliability in the Turkish population was demonstrated by Kahveci et al.^9^ We also used the NOSE score for assessment. Each parameter was evaluated individually and no difference was determined between the two groups among parameters. We found that the patients with nasal obstruction and septal deformity who undergone nasal septoplasty have very significant improvement in nasal obstruction at first month.

In our study we also evaluated the postoperative pain degree by VAS between both surgical techniques. Normally in the open septoplasty because much dissection was done in soft tissues, it is expected to have much postoperative pain.^15^ But there was no statistically significant difference in postoperative pain degrees between the two groups.

The present study has clear limitations. Major limitations of this study include the fact that only a small number of patients were surveyed and the lack of randomization. The lack of blindness could be explained obviously due to the external scar in the open technique septoplasty group.

Another limitation of our study is the use of a subjective evaluation method for comparison of different septoplasty techniques. There are many studies in the literature that evaluates the efficacy of septoplasty procedure by several more objective methods such as rhinomanometry, acoustic rhinometry or peak nasal inspiratory flow. But in previous studies the NOSE scale alone was found as effective as all those methods.^9^

Another limitation of our study is that in the endonasal technique, external deformities accompanying to septal deviation could not be fixed precisely. But in the beginning of the study we informed the patients about the outcomes and complications of both techniques and the patient selected one of them. And also in that study we only evaluate the functional results not the esthetic outcomes.

## Conclusion

We can say that the two different techniques can be performed properly in “difficult septal deviation cases” for functional result. In such difficult cases the closed septoplasty technique is at least as successful as open technique in experienced hands. The surgical technique must be chosen according to the specific conditions and the preference of the patient or the experience of the surgeon.

## Conflicts of interest

The authors declare no conflicts of interest.
